# Investigation of Er^3+^ Ions Reinforced Zinc-Phosphate Glasses for Ionizing Radiation Shielding Applications

**DOI:** 10.3390/ma14226769

**Published:** 2021-11-10

**Authors:** Hesham M. H. Zakaly, Antoaneta Ene, Oyeleke I. Olarinoye, Samir Y. Marzouk, Shams H. Abdel-Hafez, Mohamed S. Shams, Yasser S. Rammah

**Affiliations:** 1 Institute of Physics and Technology, Ural Federal University, 620002 Ekaterinburg, Russia; 2 Department of Physics, Faculty of Science, Al-Azhar University, Assiut 71524, Egypt; 3 INPOLDE Research Center, Department of Chemistry, Physics and Environment, Faculty of Sciences and Environment, Dunarea de Jos University of Galati, 47 Domneasca Street, 800008 Galati, Romania; 4 Department of Physics, School of Physical Sciences, Federal University of Technology, Minna 340110, Nigeria; iolarinoye@gmail.com; 5 Department of Basic and Applied Science, Collage of Engineering and Technology, Arab Academy of Science, Technology and Maritime Transport, AAST., Al-Horria, Heliopolis 2033, Cairo 11435, Egypt; samir_marzuk2001@yahoo.com; 6 Productivity and Quailty Institute, Arab Academy of Science, Technology and Maritime Transport, AAST., Al-Horria, Heliopolis 2033, Cairo 11435, Egypt; 7 Department of Chemistry, College of Science, Taif University, Taif 21944, Saudi Arabia; s.abdelhafz@tu.edu.sa; 8 Department of Physics and Mathematical Engineering, Faculty of Electronic Engineering, Menoufia University, Menouf 32952, Egypt; mshams_2008@yahoo.com; 9 Department of Physics, Faculty of Science, Menoufia University, Shebin El Koom 32511, Egypt; dr_yasser1974@yahoo.com

**Keywords:** phosphate glasses, photon buildup factors, fast neutron absorption, electron stopping powers

## Abstract

Melt quenching technique is used for preparing glasses with chemical formula (70P_2_O_5_)–(16 − x)CdO–(14ZnO)–(xEr_2_O_3_), (x = 1–6 mol%). These glasses were named Er1, Er2, Er3, Er4, Er5, and Er6, respectively. Photon buildup factors, fast neutron absorption, and electron stopping of the prepared glasses were examined. Glasses’ density was varied from 3.390 ± 0.003 for the Er1 glass sample to 3.412 ± 0.003 for the Er6 glass sample. The Buildup factor (BUF) spectra have relatively higher values in the Compton Scattering (CS) dominated areas compared to both Photoelectric effect (PE), and Pair Production (PP) dominated energy regions. The highest BUF appeared at the Er atom K-absorption edge, whose intensity increases as the molar concentration of Er_2_O_3_ in the glasses increases. The photon absorption efficiency (PAE) of the glasses increases according to the trend (PAE)_Er1_ < (PAE)_Er2_ < (PAE)_Er3_ < (PAE)_Er4_ < (PAE)_Er5_ < (PAE)_Er6_. Fast neutron removal cross-section, FNRC $\left(\Sigma_{R}\right)$ values of the glasses obtained via calculation varied from 0.1045–0.1039 cm^−1^ for Er1–Er6. Furthermore, the continuous slowing down approximation mode (CSDA) range enhances the kinetic energy of electrons for all glasses. Generally, results revealed that the investigated glasses could be applied for radiation shielding and dosimetric media.

## 1. Introduction

Ionizing radiation such as gamma-rays, X-rays, beta particles, protons, and neutrons have benefited modern-day humans in no small measure. As the scope of these benefits continues to rise, our understanding of different ways in which these radiations interact with atoms and molecules in biosphere and non-biological systems has widened as well. The interaction of ionizing radiation in many cases with atoms of a given medium is such that energy is exchanged. Such controlled exchange of energy within an interacting medium and its consequent effects have been found to help treat clinical symptoms such as cancer and tuning biological, physical, and chemical properties of the interacting medium [1,2,3]. On the other hand, the deleterious effect of uncontrolled radiation interaction with biological or non-living systems has made the use of radiation shields and other protective measures a significant part of radiation applications [1,3]. The choice of radiation shielding material is a function of several parameters, chief among which are the radiation type, energy, and acceptable radiation level outside the shield.

Recently, the use of different glass materials for radiation shielding has been gaining popularity [4,5,6,7]. This stems from the fact that glasses that are harsh, cheap, lightweight, radiation-resistant, non-toxic, and possess other novel characteristics can be obtained via simple synthesis methods. The choice of glass shield will continue to grow as enormous combinations of properties could be obtained via flexible and easy preparation methods. Evaluation of the shielding capacity of glass is fundamental before the glass can be deployed for shielding application. Today, due to their attractive properties, many glass compositions containing different transition metals have been investigated for their radiation shielding capacity via different experimental and theoretical procedures [4,5,6,7,8,9,10,11,12,13,14,15]. Phosphate glasses containing transition metal oxides with/without rare earth, such as Er_2_O_3,_ are characterized by high thermal expansion coefficient, high mechanical properties, low viscosity, and good optical properties [16,17,18,19].

The fact that rare-earth elements (REE) such as erbium (Er) play crucial roles in the human transition to environmentally friendly technology and economy has made glass containing Er and other REE worthy of investigation for an environmentally friendly shield for different ionizing radiations. This paper reports the effect of Er molar concentration on the photon, electron, and neutron shielding efficacy of 70P_2_O_5_.(16 − x)CdO.14ZnO.xEr_2_O_3_ glass system.

## 2. Samples Preparation and Theoretical Background

### 2.1. Samples

Six glass sample with chemical formula (70P2O5)–(16 − x)CdO–(14ZnO)–(xEr2O3), (x = 1–6 mol%) were prepared via melt quenching method. Accurate amounts of Analar grade CdO, P_2_O_5_, ZnO, and Er_2_O_3_ were combined by gently grinding all mixtures frequently to acquire a soft powder. Mix for each sample was liquefied in a small porcelain pot in an electrically warmed oven with a temperature around 950 °C–1000 °C at normal air conditions for one h to homogenize liquefy. Heat treatment is carried out for the acquired glass samples after the quenching in stainless-steel mold at around 275 °C to take off any internal thermal and mechanical stresses for 3 h. Archimedes process is used to get the studied glass samples density (ρ) using an immersion liquid such as Toluene. Table 1 shows the samples code, chemical composition, and density of the prepared glasses.

### 2.2. Photon Buildup Factors

The exponential (Beer–Lambert) equation which describes the transmission of monoenergetic and well-collimated photon beam through a thin absorbing barrier, is given in Equation (1) [1].

(1) $$I=I_{o}e^{-\mu \left(E,x\right)x}$$

where *I* and $I_{o}$ are the photon flux intensity before and after transmission through the absorbing barrier of thickness x (in a dimension of length, say, cm). The parameter μ(E,x) is called the linear attenuation coefficient (LAC) of the absorbing medium, and it depends on the thickness, x, and the photon flux energy, *E*. LAC is a measure of the number of photons that goes through the absorber without interacting. 

The measure of photon scattering by a medium in cases where the Beer–Lambert condition is not observed is estimated using the photon buildup factor (BUF). The BUF gives the correction to equation 1 in the bad geometry scenario. BUF comes under different names depending on interest’s radiation detector response function [20,21,22]. However, the energy absorption (EABUF) and exposure (EBUF) BUF are two common BUF that are generally estimated and used for shielding and dosimetric media. For EABUF and EBUF, the energy absorbed and the exposure in air equivalent thickness of the material is the detector response function, respectively. The estimation of both BUFs can be done via the very accurate Monte Carlo simulations, GP-fitting procedure, or free online software based on the GP-fitting method as the following steps [20,21,22]:

Firstly: The equivalent atomic number ($Z_{eq}$) values can be obtained with the interpolation method by proportioning the mass attenuation coefficient (MAC) values, (R) obtained for incoherent and total non-coherent interactions:

(2) $$Z_{eq}=\frac{Z_{1}\left(\log Y_{2}-\log Y\right)+Z_{2}\left(\log Y-\log Y_{1}\right)}{\log Y_{2}-\log Y_{1}}$$

where $Z_{1}$, and $Z_{2}$ are the atomic numbers related to the ratios *Y*_1_ and *Y*_2_, respectively.

Secondly: The obtained *Z_eq_* values of the investigated glasses were used to determine the GP coefficients with the next equation:

(3) $$P=\frac{P_{1}\left(\log Z_{2}-\log Z_{eq}\right)+P_{2}\left(\log Z_{eq}-\log Z_{1}\right)}{\log Z_{2}-\log Z_{1}}$$

Here *P*_1_ and *P*_2_ refer to the GP fitting parameters. Therefore, the EBF values were estimated according to the conditions in the following equations:

(4) $$B\left(E,X\right)=1+\frac{b-1}{K-1}\left(K^{x}-1\right)\;\;\;\;\;\mathrm{for}\;K\neq 1\;$$

(5) B(E,X)=1+(b−1)x       for K=1

Here,

(6) $$K\left(E,X\right)={\mathrm{Cx}}^{a}+d\frac{\tan h\left(\frac{x}{X_{k}}-2\right)-\tan h\left(-2\right)}{1-\tan h\left(-2\right)}\;\;\;\;\;\;\;\;\;\;\;\;\;\;\;\mathrm{for}\;x\leq 40$$

where K(E,X) refers to the dose multiplicative coefficient.

### 2.3. Fast Neutron Absorption

The absorption of fast (fissile) neutrons by elastic and inelastic interactions in any material is measured by a parameter called the fast neutron removal cross-section-*FNRC* ($\Sigma_{R}$). $\Sigma_{R}$ is the analogous to the linear attenuation coefficient of photons. It measures the likelihood that a fissile neutron will be removed from the fissile group on its first collision within a medium. Theoretically, $\Sigma_{R}$ of a medium can be calculated from the addition rule via Equation (7) [3,4,5,6]

(7) $$\Sigma_{R}=\underset{i}{\sum }\rho_{i}{\left(\frac{\Sigma_{R}}{\rho }\right)}_{i}$$

where ρ is the density of the medium and ${\left(\frac{\Sigma_{R}}{\rho }\right)}_{i}$ is the mass removal cross section of the *i*th element in the medium. ${\left(\frac{\Sigma_{R}}{\rho }\right)}_{i}$ is obtainable from the expressions [3]:

(8) $$\frac{\Sigma_{R}}{\rho }=0.19Z^{-0.743\;\;\;\;}\;\mathrm{for}\;Z\leq 8;\;\mathrm{and},\;\frac{\Sigma_{R}}{\rho }=0.125Z^{-0.565\;}\mathrm{for}\;Z>8$$

### 2.4. Electron Stopping Powers and Ranges

The energy loss by an electron as it moves through a material due to the coulomb (collision) and radiative losses is called the stopping power (SP). SP accounts for these losses (electronic/collision and radiative). In the continuous slowing down approximation (CSDA), the range of electrons in the material gives the average distance moved within the medium before it loses all its energy and stops [6].

Ionizing radiation interaction parameters can be determined via experimental procedure, direct calculations, and Monte Carlo simulations. Theoretical (direct) calculations have been detailed to have similar accuracy to results from experimental procedures and simulations while also having other advantages such as saving time and being cost-effective. To this end, the investigated glasses’ photon and electron shielding parameters were theoretically calculated via the free online Phy-X/PSD [22] and ESTAR [7] platforms, respectively, while the fissile neutron cross-sections were obtained via Equations (7) and (8). 

## 3. Results and Discussion

### 3.1. Photon Buildup Factors

**Figure 1:**
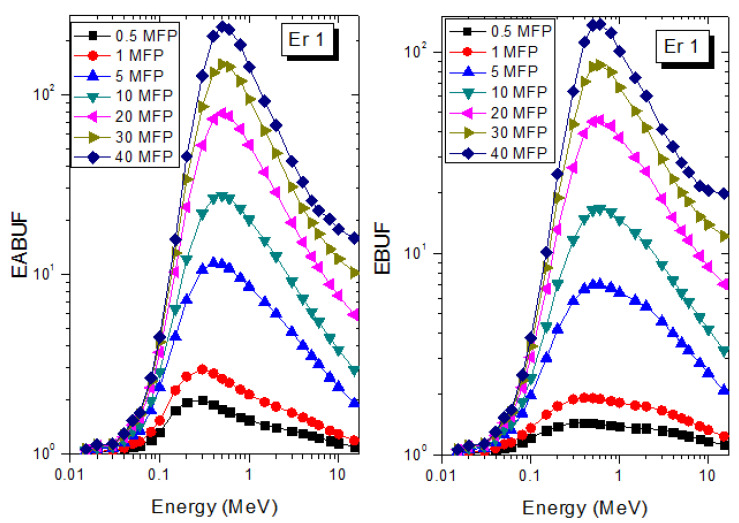
EABUF and EBUF variation with gamma-ray photon energy for Er1.

**Figure 2:**
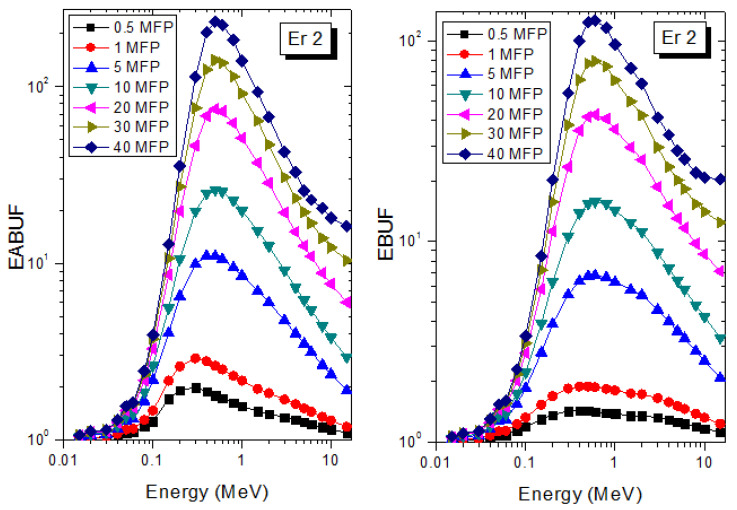
EABUF and EBUF variation with gamma-ray photon energy for Er2.

**Figure 3:**
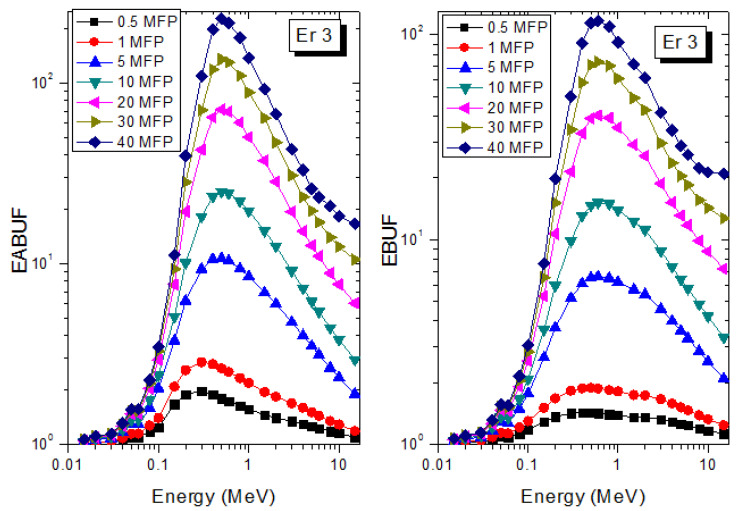
EABUF and EBUF variation with gamma-ray photon energy for Er3.

**Figure 4:**
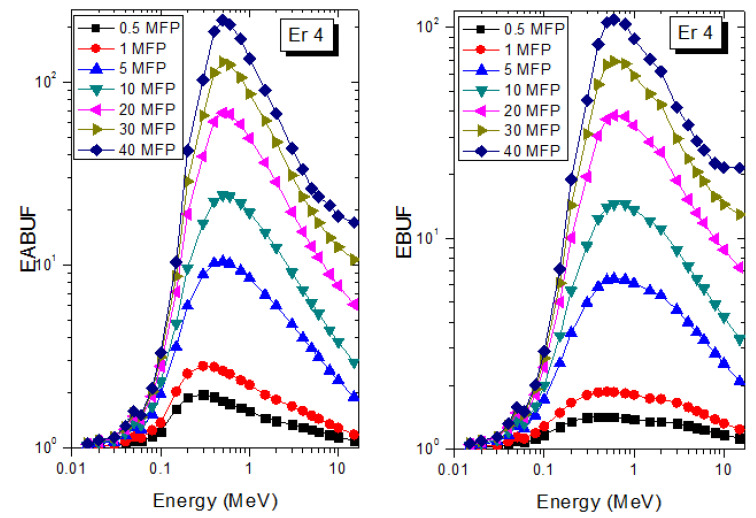
EABUF and EBUF variation with gamma-ray photon energy for Er4.

**Figure 5:**
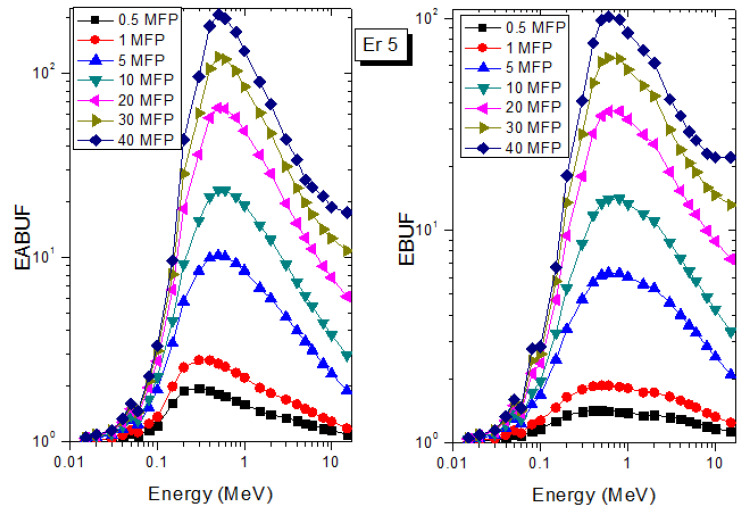
EABUF and EBUF variation with gamma-ray photon energy for Er5.

**Figure 6:**
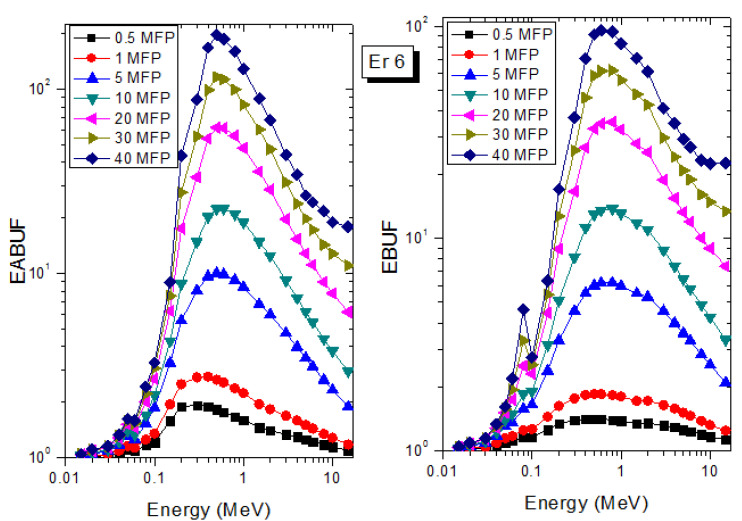
EABUF and EBUF variation with gamma-ray photon energy for Er6.

A measure of photon scattering in the glasses is evaluated via the analysis of the variation of EABUF and EBUF with photon energy, as depicted in Figure 1, Figure 2, Figure 3, Figure 4, Figure 5 and Figure 6. The figures show the pattern of changes in BUFs as photon energy changes for selected penetration depths within 40 MFP. Generally, the pattern of variation is similar for both BUFs and glass material at all the selected depths of penetration. However, one common feature of the BUF spectra has relatively higher values in the Compton scattering (CS) dominated areas compared to both photoelectric effect (PE) and pair production (PP) dominated energy regions. In fact, the trend of the magnitude of the BUFs concerning energy regions where each of these interactions dominates is (BUF)_PE_ < (BUF)_PP_ < (BUF)_CS_. This trend is consistent with the fact that both PE and PP are processes that lead to total photon absorption while CS scatters the photon; thus, BUF is high in CS region. Furthermore, the annihilation of electron–positron pairs created by the PP process produces photons whenever higher photon buildup occurs in the PP region compared to the PE region [20]. The G–P fitting coefficients (b, c, a, X_k_, and d) of Er1–Er6 samples for EBF and EABF are tabulated in Supplementary Tables S1–S6, respectively. 

**Figure 7:**
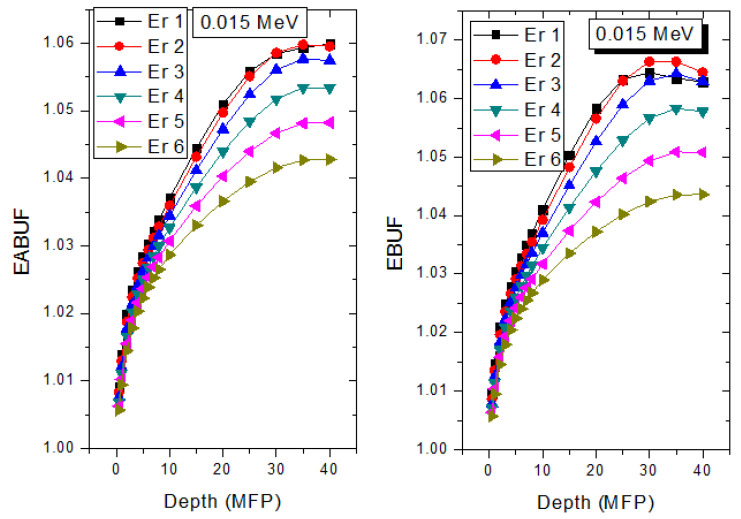
Effect of chemical composition on EABUF and EBUF at different depth for 0.015 MeV photon energy.

**Figure 8:**
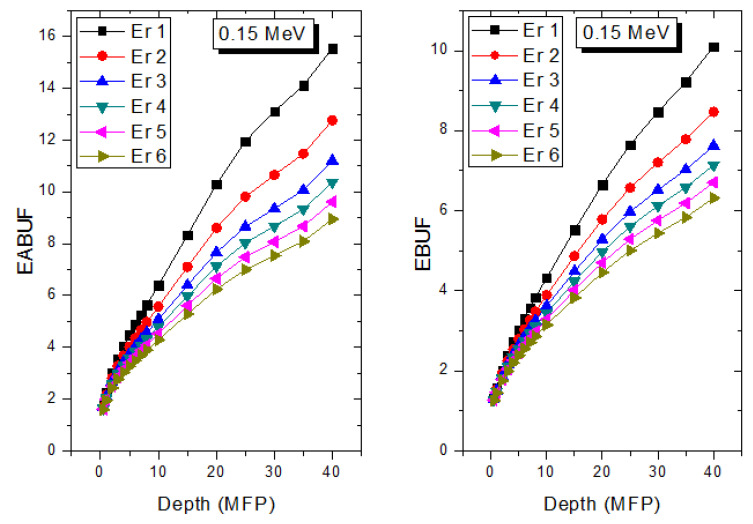
Effect of chemical composition on EABUF and EBUF at different depth for 0.15 MeV photon energy.

**Figure 9:**
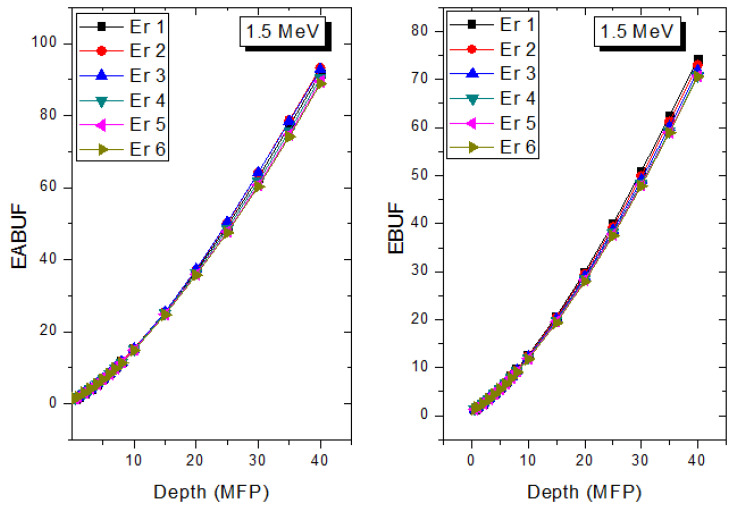
Effect of chemical composition on EABUF and EBUF at different depth for 1.5 MeV photon energy.

**Figure 10:**
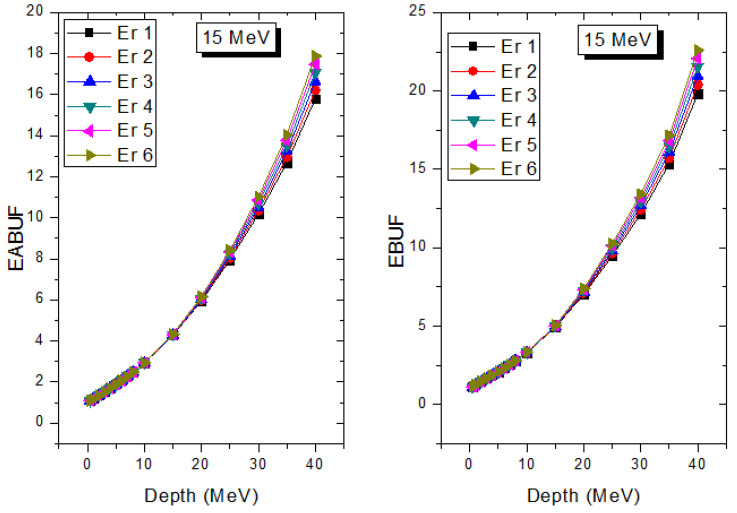
Effect of chemical composition on EABUF and EBUF at different depths for 15 MeV photon energy.

The peak of both EABUF and EBUF appeared at a photon energy of 0.5 MeV for all the glasses. Another notable feature of the BUF spectra is the appearance of high BUF at the Er atom K-absorption edge, whose intensity increase as the molar concentration of Er_2_O_3_ in the glasses increase. This is due to the fluorescence that takes place after the K-electron photon absorption. A BUF greater than unity at a depth of 0.5 MFP in the glasses shows that the optimum glass thickness to prevent photon scattering in the glasses is below an equivalent thickness of 0.5 MFP of all the glasses. In order to investigate the effect of the chemical composition of the glasses on their photon scattering capacity, EABUF and EBUF of the glasses were plotted against depth at selected photon energies (0.015, 0.15, 1.5, and 15 MeV) and presented in Figure 7, Figure 8, Figure 9 and Figure 10. At 0.015 MeV, the BUF is low due to the absorption of a photon by the PE process, while at 0.15 and 1.5 MeV, the BUFs increase in magnitude. Among the compared energies, BUF is maximum for 0.015 MeV and maximum for 1.5 MeV, in agreement with the effect of PE, CS, and PP. Figures also show a consistent trend in the increase in BUF as the mass density and Er_2_O_3_ reduce in the glasses. This shows that photon absorption efficiency (PAE) of the glasses increases according to the trend (PAE)_Er1_ < (PAE)_Er2_ < (PAE)_Er3_ < (PAE)_Er4_ < (PAE)_Er5_ < (PAE)_Er6._ This also affirms the fact that PEA of a material depends on the photon energy and chemical composition of the shielding material.

### 3.2. Fast Neutron Absorption

**Figure 11:**
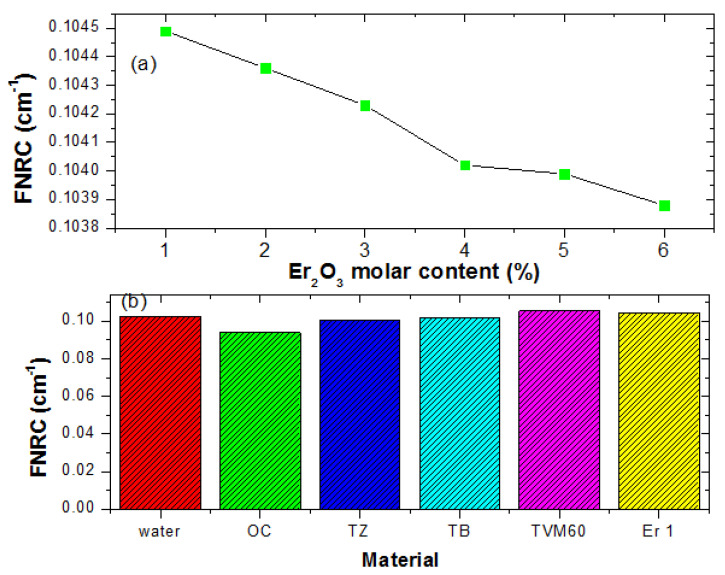
Influence of Er_2_O_3_ molar concentration on the FNRC of the glasses (**a**) and FNRC of Er1 compared to other materials (**b**).

Fast neutron removal cross-section, FNRC $\left(\Sigma_{R}\right)$ values of the glasses obtained via calculation varied from 0.1045–0.1039 cm^−1^ for Er1–Er6 glasses. A consistent decrease in the value of ${\;\Sigma }_{R}$ is pictorially represented in Figure 11a. Obviously, ${\;\Sigma }_{R}$ decrease with an increase in the Er_2_O_3_ content of the glasses. This is due to the higher fast neutron microscopic removal cross section of Cd compared to Er. Hence as the Cd content decreases and Er increases in the glasses, FNRC declines in magnitude. A comparison of FNRC of Er1 with those of water, OC, and recently developed glass systems- TB, TZ, and TVM60 [23,24,25] is presented in Figure 11b. It is evident that Er1 is a better fast neutron absorber compared to these materials except TVM60.

### 3.3. Stopping Powers (S_e_) and Range of Electrons

**Figure 12:**
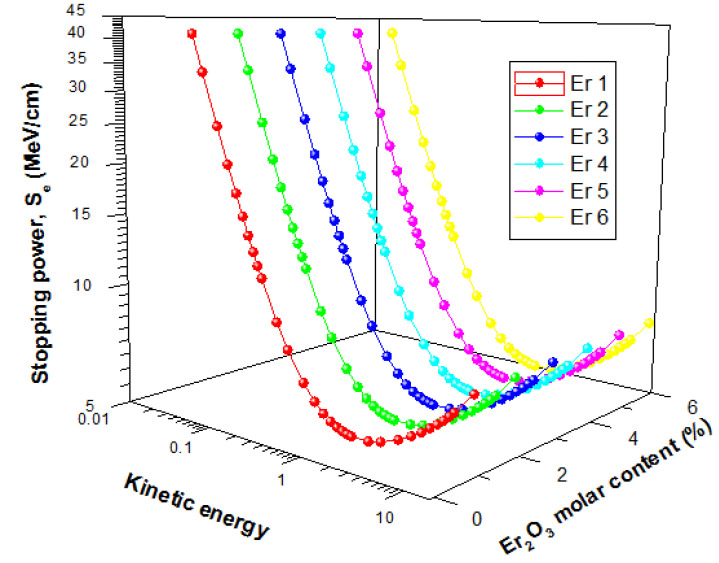
Stopping powers of Electron in the glasses as a function of kinetic energy.

The energetic electron shielding capacity of the present glasses was investigated using stopping powers (S_e_) and Range (continuous slowing down approximation mode (CSDA)) data for kinetic energies within 15 MeV. Figure 12 displays the changes in the value of S_e_ and Range of the glasses as a function of electron kinetic energy.

**Figure 13:**
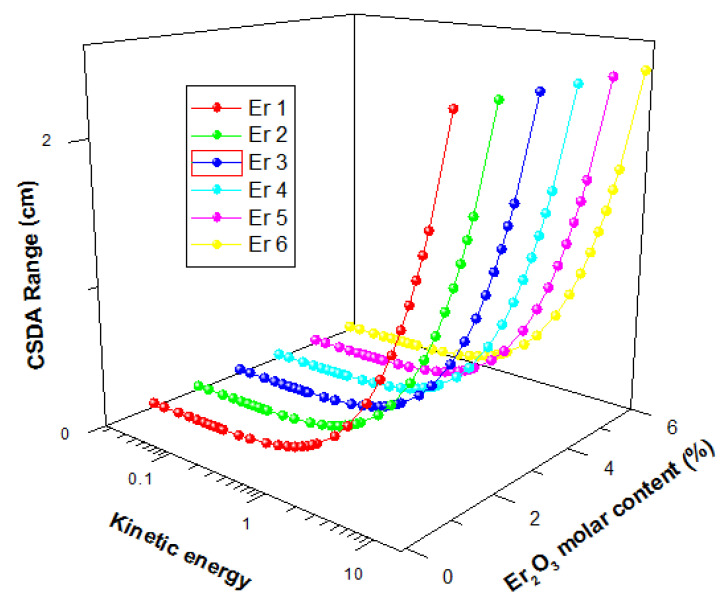
CSDA range of electrons in the glasses as a function of kinetic energy.

The figure shows that there was a general initial decrease of S_e_ with energy up to an energy of 1 MeV before increasing with energy further. The initial decrease is due to collision losses that decrease with energy, while the latter increase in Se is attributed to energy losses via radiation losses. Radiation yield of energetic electron increase with energy hence the increase in S_e_ beyond 1 MeV with kinetic energy. For the glasses, S_e_ values were very close, with insignificant differences. The CSDA range increase with a kinetic energy of Electron for all glasses, as shown in Figure 13. An increase in the kinetic energy of an electron leads to an increase in its penetrating ability. Hence the observed increase in Range with kinetic energy. Similar to the S_e_, there was no significant difference between the Range of electrons in the glasses. Hence, an increase in Er_2_O_3_ content of the glasses within the molar concentration considered in this study does not significantly affect the electron absorption capacity of presently studied glasses.

## 4. Conclusions

Melt quenching technique is used for preparing glasses with chemical formula (70P_2_O_5_)–(16 − x)CdO–(14ZnO)–(xEr_2_O_3_), (x = 1–6 mol%). These glasses were named Er1, Er2, Er3, Er4, Er5, and Er6, respectively. Photon buildup factors, fast neutron absorption, and electron stopping of the prepared glasses were examined. Results revealed that:

1- Glasses’ density was varied from 3.390 ± 0.003 for Er1 glass sample to 3.412 ± 0.003 for E6 glass sample.
2- The BUF spectra have relatively higher values in the Compton Scattering (CS) dominated areas compared to both photoelectric effect (PE), and Pair Production (PP) dominated energy regions.
3- The highest BUF appeared at the Er atom K-absorption edge, whose intensity increases as the molar concentration of Er_2_O_3_ in the glasses increases.
4- The photon absorption efficiency (PAE) of the glasses increases according to the trend (PAE)_Er1_ < (PAE)_Er2_ < (PAE)_Er3_ < (PAE)_Er4_ < (PAE)_Er5_ < (PAE)_Er6_.
5- Fast neutron removal cross-section, FNRC $\left(\;\Sigma_{R}\right)$ values of the glasses obtained via calculation varied from 0.1045–0.1039 cm^−1^ for Er1–Er6.
6- The continuous slowing down approximation mode (CSDA) range increases with a kinetic energy of electrons for all the investigated glasses.

Therefore, the suggested glasses can be used for radiation shielding and dosimetric media.

## Figures and Tables

**Table 1:** Glasses code, composition, density of the prepared glasses (70P_2_O_5_)–(16 − x)CdO–(14ZnO)–(xEr_2_O_3_), (x = 1–6 mol%).

Glass	Composition mol%				Density (gm/cm^3^) ± 0.003
	ZnO	CdO	P_2_O_5_	Er_2_O_3_	
Er1	14	15	70	1	3.390
Er2	14	14	70	2	3.395
Er3	14	13	70	3	3.399
Er4	14	12	70	4	3.403
Er5	14	11	70	5	3.408
Er6	14	10	70	6	3.412

## Data Availability

Data is contained within the article.

## Supplementary Materials

The following are available online at https://www.mdpi.com/article/10.3390/ma14226769/s1, Table S1: (EBF and EABF) G–P fitting coefficients (b, c, a, Xk and d) of Er1 sample, Table S2: (EBF and EABF) G–P fitting coefficients (b, c, a, Xk and d) of Er2 sample, Table S3: (EBF and EABF) G–P fitting coefficients (b, c, a, Xk and d) of Er3 sample, Table S4: (EBF and EABF) G–P fitting coefficients (b, c, a, Xk and d) of Er4 sample, Table S5: (EBF and EABF) G–P fitting coefficients (b, c, a, Xk and d) of Er5 sample, Table S6: (EBF and EABF) G–P fitting coefficients (b, c, a, Xk and d) of Er6 sample.
