# Supplementary material for: Investigation of Er3+ Ions Reinforced Zinc-Phosphate Glasses for Ionizing Radiation Shielding Applications
Source: Materials (Basel). 2021 Nov 10;14(22):6769. doi: 10.3390/ma14226769 (PMC8618482; doi:10.3390/ma14226769)
Supplement: Supplementary file 1 [file materials-14-06769-s001.zip › materials-1434596-supplementary.pdf]

Table S1: (EBF and EABF) G–P fitting coefficients (b, c, a, Xk and d) of Er1 sample.

	G-P Fitting Parameters for EBF					*G-P Fitting Parameters for EABF*				
**Energy** *MeV*	**a**	**b**	**c**	**d**	**Xk**	**a**	**b**	**c**	**d**	**Xk**
0.015	0.272	1.015	0.351	-0.262	15.855	0.256	1.014	0.351	-0.198	16.512
0.02	0.239	1.034	0.330	-0.299	23.931	0.214	1.032	0.374	-0.417	29.484
0.03	0.204	1.040	0.373	-0.238	22.243	0.246	1.039	0.336	-0.173	16.459
0.04	0.244	1.079	0.344	-0.124	12.353	0.240	1.078	0.345	-0.128	13.904
0.05	0.228	1.127	0.375	-0.131	14.039	0.239	1.135	0.354	-0.135	14.360
0.06	0.208	1.150	0.405	-0.114	14.171	0.229	1.169	0.373	-0.136	14.701
0.08	0.182	1.253	0.466	-0.100	14.387	0.196	1.323	0.433	-0.111	14.979
0.1	0.151	1.356	0.541	-0.083	14.039	0.163	1.529	0.502	-0.099	16.432
0.15	0.090	1.583	0.706	-0.052	14.049	0.182	2.257	0.523	-0.129	14.183
0.2	0.046	1.741	0.859	-0.035	13.496	0.107	2.693	0.708	-0.086	13.345
0.3	0.003	1.883	1.035	-0.019	12.402	0.034	2.944	0.940	-0.047	12.745
0.4	-0.019	1.914	1.139	-0.014	11.405	0.000	2.797	1.077	-0.032	12.152
0.5	-0.029	1.910	1.190	-0.011	9.894	-0.020	2.612	1.159	-0.019	11.540
0.6	-0.035	1.888	1.215	-0.009	8.560	-0.028	2.480	1.189	-0.015	10.757
0.8	-0.037	1.846	1.224	-0.012	7.142	-0.032	2.274	1.206	-0.014	8.868
1	-0.034	1.815	1.202	-0.011	7.582	-0.035	2.134	1.207	-0.009	7.251
1.5	-0.040	1.754	1.198	0.011	15.888	-0.040	1.938	1.196	0.010	15.372
2	-0.030	1.737	1.148	0.008	17.383	-0.029	1.834	1.144	0.007	17.360
3	-0.006	1.657	1.052	-0.009	11.536	-0.007	1.692	1.052	-0.006	12.848
4	0.008	1.582	1.002	-0.015	10.717	0.011	1.592	0.989	-0.018	12.734
5	0.014	1.514	0.978	-0.019	12.880	0.017	1.506	0.966	-0.027	15.193
6	0.020	1.465	0.961	-0.023	13.573	0.024	1.441	0.949	-0.031	14.162
8	0.031	1.385	0.931	-0.032	13.414	0.031	1.348	0.928	-0.029	12.775
10	0.036	1.324	0.919	-0.036	13.615	0.040	1.287	0.909	-0.039	13.598
15	0.056	1.233	0.880	-0.055	13.445	0.036	1.185	0.930	-0.038	14.334

Table S2: (EBF and EABF) G–P fitting coefficients (b, c, a, Xk and d) of Er2 sample.

Energy	*G-P Fitting Parameters for EBF*					*G-P Fitting Parameters for EABF*				
MeV	**a**	**b**	**c**	**d**	**Xk**	**a**	**b**	**c**	**d**	**Xk**
0.015	0.246	1.014	0.380	-0.266	18.603	0.233	1.013	0.381	-0.215	19.126
0.02	0.242	1.032	0.327	-0.270	22.558	0.197	1.029	0.399	-0.386	29.785
0.03	0.205	1.040	0.373	-0.233	21.798	0.246	1.039	0.337	-0.172	16.307
0.04	0.244	1.081	0.345	-0.124	12.418	0.240	1.080	0.347	-0.128	13.901
0.05	0.227	1.131	0.376	-0.131	14.042	0.239	1.139	0.355	-0.135	14.355
0.06	0.216	1.138	0.394	-0.120	14.004	0.235	1.155	0.364	-0.140	14.655
0.08	0.186	1.229	0.458	-0.103	14.233	0.204	1.295	0.419	-0.115	14.762
0.1	0.157	1.324	0.525	-0.088	13.970	0.149	1.460	0.519	-0.094	17.818
0.15	0.097	1.535	0.683	-0.055	14.006	0.201	2.156	0.486	-0.135	13.689
0.2	0.053	1.693	0.833	-0.037	13.625	0.121	2.601	0.668	-0.094	13.454
0.3	0.009	1.849	1.006	-0.021	12.623	0.046	2.895	0.900	-0.052	12.872
0.4	-0.013	1.891	1.114	-0.015	11.697	0.011	2.785	1.039	-0.038	12.340
0.5	-0.024	1.895	1.167	-0.012	10.384	-0.011	2.621	1.124	-0.024	11.806
0.6	-0.030	1.878	1.194	-0.010	9.163	-0.019	2.499	1.156	-0.019	11.093
0.8	-0.033	1.841	1.206	-0.012	7.929	-0.026	2.300	1.179	-0.017	9.417
1	-0.031	1.812	1.189	-0.011	8.257	-0.029	2.161	1.183	-0.012	7.978
1.5	-0.039	1.746	1.194	0.011	16.451	-0.036	1.941	1.183	0.006	13.743
2	-0.029	1.733	1.144	0.006	15.976	-0.029	1.833	1.143	0.007	18.560
3	-0.005	1.657	1.050	-0.009	11.511	-0.007	1.690	1.054	-0.006	12.735
4	0.007	1.580	1.003	-0.015	10.846	0.012	1.592	0.987	-0.019	12.645
5	0.014	1.514	0.978	-0.020	13.049	0.017	1.505	0.968	-0.028	15.341
6	0.019	1.464	0.963	-0.023	13.608	0.025	1.440	0.947	-0.032	13.857
8	0.030	1.384	0.933	-0.032	13.409	0.032	1.346	0.927	-0.030	12.668
10	0.037	1.323	0.919	-0.037	13.577	0.040	1.286	0.909	-0.040	13.659
15	0.059	1.235	0.873	-0.058	13.523	0.036	1.184	0.934	-0.038	14.370

Table S3: (EBF and EABF) G–P fitting coefficients (b, c, a, Xk and d) of Er3 sample.

	G-P Fitting Parameters for EBF					*G-P Fitting Parameters for EABF*				
Energy MeV	**a**	**b**	**c**	**d**	**Xk**	**a**	**b**	**c**	**d**	**Xk**
0.015	0.220	1.012	0.408	-0.271	21.208	0.210	1.012	0.408	-0.232	21.605
0.02	0.246	1.030	0.324	-0.242	21.253	0.181	1.027	0.422	-0.356	30.071
0.03	0.206	1.041	0.373	-0.228	21.344	0.245	1.040	0.338	-0.171	16.152
0.04	0.243	1.082	0.346	-0.125	12.483	0.239	1.082	0.348	-0.128	13.899
0.05	0.227	1.134	0.377	-0.130	14.046	0.238	1.142	0.356	-0.135	14.350
0.06	0.223	1.128	0.384	-0.125	13.843	0.240	1.143	0.355	-0.144	14.612
0.08	0.189	1.208	0.452	-0.106	14.096	0.211	1.269	0.407	-0.119	14.567
0.1	0.163	1.295	0.511	-0.091	13.908	0.137	1.399	0.534	-0.089	19.053
0.15	0.104	1.504	0.666	-0.057	14.012	0.212	2.086	0.464	-0.138	13.540
0.2	0.064	1.674	0.804	-0.042	13.684	0.139	2.572	0.636	-0.103	13.506
0.3	0.016	1.824	0.980	-0.023	12.806	0.057	2.845	0.867	-0.057	12.989
0.4	-0.008	1.872	1.091	-0.017	11.954	0.021	2.774	1.005	-0.043	12.502
0.5	-0.020	1.882	1.147	-0.013	10.804	-0.003	2.629	1.092	-0.028	12.029
0.6	-0.027	1.869	1.175	-0.011	9.683	-0.012	2.516	1.127	-0.023	11.384
0.8	-0.030	1.837	1.191	-0.012	8.611	-0.020	2.323	1.155	-0.019	9.891
1	-0.029	1.809	1.178	-0.011	8.840	-0.025	2.184	1.163	-0.014	8.606
1.5	-0.038	1.740	1.190	0.011	17.397	-0.030	1.947	1.166	-0.001	10.987
2	-0.027	1.729	1.140	0.004	14.668	-0.028	1.832	1.141	0.007	19.676
3	-0.005	1.658	1.048	-0.010	11.362	-0.008	1.687	1.056	-0.006	12.497
4	0.007	1.579	1.005	-0.014	10.970	0.013	1.592	0.985	-0.020	12.560
5	0.015	1.513	0.978	-0.021	13.213	0.017	1.503	0.969	-0.028	15.484
6	0.019	1.463	0.964	-0.023	13.641	0.026	1.439	0.946	-0.032	13.559
8	0.030	1.383	0.934	-0.033	13.404	0.033	1.345	0.926	-0.031	12.562
10	0.037	1.323	0.919	-0.038	13.539	0.040	1.284	0.910	-0.041	13.720
15	0.062	1.237	0.866	-0.061	13.600	0.035	1.182	0.937	-0.037	14.406

Table S4: (EBF and EABF) G–P fitting coefficients (b, c, a, Xk and d) of Er4 sample.

	G-P Fitting Parameters for EBF					*G-P Fitting Parameters for EABF*				
Energy MeV	**a**	**b**	**c**	**d**	**Xk**	**a**	**b**	**c**	**d**	**Xk**
0.015	0.196	1.011	0.435	-0.275	23.726	0.189	1.011	0.435	-0.249	24.001
0.02	0.249	1.029	0.321	-0.216	20.045	0.166	1.024	0.444	-0.329	30.335
0.03	0.207	1.042	0.373	-0.223	20.887	0.245	1.041	0.339	-0.170	15.996
0.04	0.243	1.084	0.346	-0.125	12.548	0.238	1.084	0.349	-0.128	13.897
0.05	0.226	1.138	0.378	-0.130	14.050	0.238	1.146	0.358	-0.135	14.345
0.06	0.230	1.119	0.374	-0.130	13.695	0.245	1.132	0.348	-0.147	14.572
0.08	0.192	1.189	0.446	-0.109	13.971	0.217	1.246	0.395	-0.123	14.391
0.1	0.171	1.281	0.497	-0.096	13.883	0.143	1.372	0.525	-0.091	19.142
0.15	0.109	1.482	0.651	-0.060	14.045	0.218	2.034	0.453	-0.140	13.586
0.2	0.073	1.656	0.779	-0.047	13.737	0.155	2.546	0.607	-0.111	13.552
0.3	0.022	1.801	0.956	-0.025	12.968	0.067	2.801	0.837	-0.061	13.091
0.4	-0.003	1.856	1.070	-0.019	12.179	0.030	2.764	0.976	-0.047	12.645
0.5	-0.016	1.870	1.129	-0.014	11.175	0.005	2.636	1.065	-0.032	12.226
0.6	-0.023	1.861	1.159	-0.012	10.140	-0.006	2.530	1.102	-0.026	11.639
0.8	-0.028	1.834	1.178	-0.012	9.206	-0.015	2.343	1.134	-0.021	10.306
1	-0.027	1.806	1.169	-0.011	9.351	-0.020	2.205	1.145	-0.016	9.156
1.5	-0.037	1.734	1.186	0.011	18.232	-0.026	1.951	1.151	-0.008	8.552
2	-0.026	1.726	1.137	0.002	13.434	-0.028	1.831	1.140	0.007	20.729
3	-0.005	1.655	1.051	-0.010	11.573	-0.008	1.685	1.056	-0.006	12.288
4	0.008	1.579	1.004	-0.016	11.174	0.014	1.591	0.984	-0.021	12.321
5	0.015	1.513	0.978	-0.021	13.372	0.017	1.501	0.970	-0.029	15.623
6	0.019	1.462	0.965	-0.023	13.674	0.026	1.438	0.945	-0.033	13.270
8	0.030	1.382	0.936	-0.033	13.399	0.034	1.344	0.926	-0.032	12.459
10	0.038	1.322	0.919	-0.039	13.502	0.040	1.283	0.910	-0.042	13.779
15	0.065	1.239	0.859	-0.064	13.676	0.035	1.180	0.940	-0.037	14.441

Table S5: (EBF and EABF) G–P fitting coefficients (b, c, a, Xk and d) of Er5 sample.

	G-P Fitting Parameters for EBF					*G-P Fitting Parameters for EABF*				
Energy MeV	**a**	**b**	**c**	**d**	**Xk**	**a**	**b**	**c**	**d**	**Xk**
0.015	0.173	1.010	0.460	-0.279	26.071	0.169	1.010	0.460	-0.264	26.232
0.02	0.252	1.027	0.318	-0.192	18.924	0.152	1.022	0.464	-0.303	30.580
0.03	0.208	1.042	0.373	-0.218	20.434	0.245	1.041	0.340	-0.169	15.841
0.04	0.242	1.086	0.347	-0.126	12.616	0.238	1.086	0.351	-0.127	13.894
0.05	0.226	1.141	0.379	-0.129	14.053	0.237	1.150	0.359	-0.135	14.340
0.06	0.236	1.110	0.365	-0.134	13.560	0.249	1.122	0.341	-0.150	14.535
0.08	0.229	1.220	0.419	-0.119	13.998	0.240	1.250	0.375	-0.129	14.367
0.1	0.180	1.272	0.483	-0.100	13.874	0.156	1.359	0.506	-0.097	18.764
0.15	0.115	1.463	0.638	-0.062	14.075	0.224	1.986	0.442	-0.142	13.627
0.2	0.082	1.640	0.756	-0.052	13.785	0.169	2.523	0.581	-0.119	13.593
0.3	0.028	1.781	0.935	-0.027	13.113	0.076	2.762	0.811	-0.064	13.184
0.4	0.001	1.841	1.052	-0.020	12.381	0.037	2.755	0.950	-0.051	12.772
0.5	-0.013	1.860	1.113	-0.015	11.506	0.011	2.642	1.040	-0.035	12.401
0.6	-0.020	1.854	1.144	-0.013	10.549	0.000	2.544	1.079	-0.029	11.867
0.8	-0.026	1.830	1.167	-0.012	9.737	-0.011	2.361	1.116	-0.023	10.676
1	-0.026	1.803	1.160	-0.011	9.805	-0.016	2.223	1.129	-0.018	9.645
1.5	-0.036	1.725	1.183	0.010	17.526	-0.024	1.951	1.145	-0.009	8.573
2	-0.025	1.723	1.134	0.000	12.268	-0.027	1.830	1.139	0.007	21.723
3	-0.008	1.648	1.060	-0.008	12.392	-0.006	1.686	1.053	-0.008	12.122
4	0.008	1.579	1.003	-0.017	11.372	0.014	1.590	0.984	-0.023	12.090
5	0.014	1.512	0.980	-0.021	13.389	0.019	1.501	0.966	-0.031	14.867
6	0.019	1.461	0.966	-0.024	13.686	0.027	1.437	0.944	-0.033	13.027
8	0.030	1.381	0.937	-0.033	13.394	0.034	1.343	0.925	-0.033	12.360
10	0.038	1.322	0.919	-0.040	13.466	0.041	1.282	0.911	-0.042	13.836
15	0.067	1.241	0.853	-0.066	13.749	0.034	1.179	0.943	-0.037	14.474

Table S6: (EBF and EABF) G–P fitting coefficients (b, c, a, Xk and d) of Er6 sample.

	G-P Fitting Parameters for EBF					*G-P Fitting Parameters for EABF*				
Energy MeV	**a**	**b**	**c**	**d**	**Xk**	**a**	**b**	**c**	**d**	**Xk**
0.015	0.152	1.009	0.483	-0.283	28.263	0.150	1.009	0.483	-0.278	28.317
0.02	0.262	1.026	0.310	-0.179	17.190	0.207	1.022	0.409	-0.289	25.857
0.03	0.209	1.043	0.373	-0.212	19.972	0.244	1.042	0.342	-0.167	15.684
0.04	0.242	1.088	0.348	-0.126	12.684	0.237	1.087	0.352	-0.127	13.892
0.05	0.225	1.145	0.380	-0.129	14.057	0.237	1.154	0.360	-0.134	14.335
0.06	0.279	1.172	0.346	-0.136	13.734	0.277	1.131	0.325	-0.152	14.549
0.08	0.264	1.248	0.394	-0.128	14.024	0.262	1.253	0.356	-0.136	14.346
0.1	0.187	1.264	0.470	-0.104	13.865	0.168	1.348	0.488	-0.102	18.416
0.15	0.119	1.445	0.627	-0.065	14.103	0.229	1.944	0.432	-0.143	13.665
0.2	0.090	1.626	0.735	-0.056	13.829	0.182	2.502	0.557	-0.126	13.631
0.3	0.033	1.763	0.915	-0.028	13.244	0.083	2.726	0.787	-0.067	13.267
0.4	0.005	1.828	1.036	-0.022	12.563	0.044	2.748	0.926	-0.055	12.887
0.5	-0.010	1.851	1.099	-0.016	11.805	0.017	2.648	1.018	-0.038	12.560
0.6	-0.018	1.848	1.131	-0.013	10.919	0.005	2.555	1.059	-0.032	12.073
0.8	-0.024	1.827	1.156	-0.012	10.215	-0.007	2.377	1.099	-0.025	11.009
1	-0.024	1.801	1.152	-0.011	10.213	-0.013	2.239	1.114	-0.019	10.085
1.5	-0.036	1.716	1.181	0.009	16.618	-0.023	1.950	1.140	-0.009	9.000
2	-0.024	1.720	1.131	-0.001	11.180	-0.027	1.829	1.138	0.007	22.652
3	-0.011	1.641	1.069	-0.007	13.185	-0.005	1.687	1.050	-0.010	11.961
4	0.009	1.579	1.002	-0.018	11.563	0.015	1.589	0.983	-0.024	11.872
5	0.014	1.510	0.983	-0.021	13.398	0.021	1.501	0.962	-0.033	14.088
6	0.020	1.462	0.963	-0.025	13.608	0.027	1.435	0.944	-0.034	12.954
8	0.030	1.380	0.938	-0.034	13.394	0.035	1.342	0.924	-0.034	12.272
10	0.039	1.321	0.919	-0.041	13.431	0.041	1.280	0.911	-0.043	13.892
15	0.070	1.243	0.846	-0.069	13.820	0.033	1.177	0.946	-0.037	14.507
